# An Account of Immune Senescence in the Clinical Pathophysiology of COVID-19 Infection in Aging

**DOI:** 10.14336/AD.2020.1019

**Published:** 2021-04-01

**Authors:** Shilpi Jain, Eden Abrham, M. Nadeem Khan, Ramkumar Mathur

**Affiliations:** ^1^Department of Geriatrics, University of North Dakota, Grand Forks, North Dakota 58202, USA; ^2^Department of Biomedical Science, University of North Dakota, Grand Forks, North Dakota 58202, USA

**Keywords:** COVID-19, SARS-CoV-2, aging, immune senescence, cytokine storm, therapeutics

## Abstract

Worldwide COVID-19 infection poses an enormous risk to public health and an alarming global socioeconomic burden. The impact of the COVID-19 pandemic on individuals with underlying health conditions as well as on the elderly population is extensive and effective strategies are needed to understand the mechanism behind it. Cellular senescence defines as an irreversible cell cycle arrest due to DNA damage leading to accumulation of senescent cells in the elderly population and may result in worsening of COVID-19 mediated increased mortality. However, whether this variation in senescence levels, in different aged populations, translation to COVID-19 infection is unknown. The spike protein of SARS-CoV-2 has been recently identified to be responsible for inducing pathogenic signals, although a clear understanding of how the host receptor interacts with SARS-CoV-2 protein and mediates the immune responses is not clear. In this review, we address the epidemiology of SARS-CoV-2 and the cellular senescence responding immune response to pathogenic SARS-CoV-2. We provide a prospective summary of what to expect and how to brace the possible immunological strategy to protect against COVID-19 infection. The review majorly explores an underline mechanism of how senescent cells trigger a hyperimmune inflammatory response and cause high mortality in aging people could serve as a potential aid to alleviate the treatment for elderly battling COVID-19 infection.

## 1. COVID-19 pandemic and the aging community

**Figure 1.**
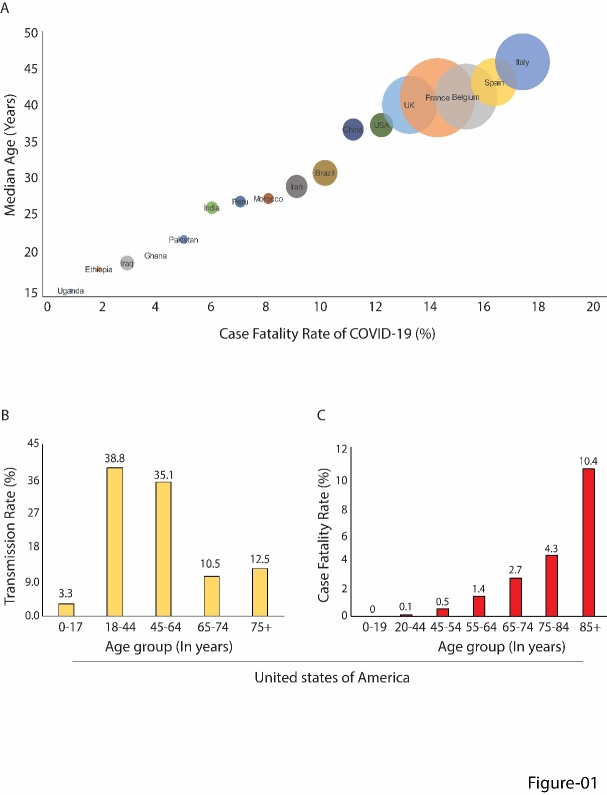
A correlation between aging and COVID-fatality. (A) Data from different countries showing a correlation between median age and case fatality rate of COVID-19. The case fatality rate calculated by dividing confirmed death with the total number of cases (source: https://ourworldindata.org/grapher/case-fatality-rate-of-covid-19-vs-median-age). (B) The representative data shows the SARS-CoV-2 transmission rate and (C) the fatality rate in the United States (source: www.cdc.gov/coronavirus/2019-ncov/casesupdates/cases-in-us.html); where the case fatality rate and hospitalization rate successively increase with aging [2].

The rapid transmission of SARS-CoV-2 (Severe Acute Respiratory Syndrome Corona Virus-2) lately emerges as an nCOVID-19 pandemic, 37.7 million confirmed cases, 1 million fatalities, and massive socio-economic burdens, as of the writing of this article [1]. With 7.8 million SARS-CoV-2 positive cases and 215,022 deaths, the United States is the worst affected nation with COVID-19 infection [2]. The COVID-19 situation is declared a pandemic by World Health Organization (WHO) on March 11, 2020. The most common symptoms are headache, loss of smell, nasal obstruction, cough, myalgia, sore throat, congestion, and diarrhea in early phase of COVID-19 infection. Most clinical cases found to resolve within 7 days of infection. However, Acute Respiratory Distress Syndrome (ARDS) and acute respiratory failure complication develops in advance phase of COVID-19 infection, which requires mechanical ventilation support to patients admitted in the ICU [3-5].Individuals underlying medical conditions such as cardiovascular disease, diabetes mellitus, cancer, or advanced chronological aging perturb a high number of co-morbidities in COVID-19 infection [6, 7]. Remarkably, the countries having high median age populations found increased disease burden and high fatality rates compared to the country having low median age population, suggesting a positive correlation between the increased age and case fatality rate (Fig. 1A). According to a report released by Centers for Disease Control and Prevention (CDC), fatality was highest in the ≥85 age group ranging from 10% to 27% as compared to 3% to 11% in 65-84, 1% to 3% 55-64 years age group, <1% in 20-54 aged, and no mortalities persons aged below 19 years in the United States. Interestingly, the transmission rate of SARS-CoV-2 found comparable to the young age and elderly population in representative data of the United States; however, the statistics showed that the fatality rate is successively corresponding to aging (Fig. 1B). It indicates that the compromised immune system in the aging population is not enough to clear SARS-CoV-2 transmission besides less transmission and leads to much higher mortality in the elderly. As this outbreak identifies older age as a high risk of mortality and co-morbidities, an underlying mechanistic study with emphasis on advanced chronological age persons is required to find an effective clinical outcome of COVID-19 infection as predicted by the estimation of severity of COVID-19 [8-10].

**Figure 2.**
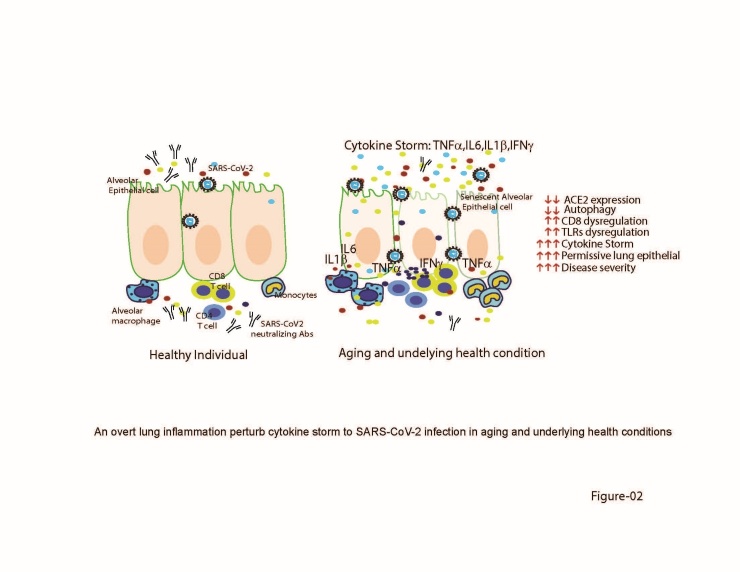
An overt lung inflammation perturb cytokine storm to SARS-CoV-2 infection in aging underlying health condition. Senescence alveolar lung epithelial cells from aging and underlying health conditions individuals exhibit, decline autophagy, low ACE2, and dysregulated innate and adaptive CD4, CD8 cells produce, which produces a high amount of IFNγ, IL6, TNFα cytokine in aging populations. The increased amount of cytokine storm level consequences in higher co-morbidities in aging and underlying health condition individuals.

## 2. Comparative etiology of SARS-CoV-2 and cross-species viral transmission

Coronaviruses are defined as zoonotic, enveloped, and large 27.9-31 kb positive-stranded RNA viruses, which belong to the *Coronaviridae* family [11]. Based on encoded protein sequences, CoVs classified as alpha-CoV, beta-CoV, gamma-CoV, and delta-CoV [12]. β-coronaviruses contain highly pathogenic SARS-CoV-2 [13], SARS-CoV, and Middle East Respiratory Syndrome (MERS)-CoV all involved in preceding fatal outbreaks [14, 15]. Cross-species viral pathogenesis is found to be the cause of emergence of many new viruses responsible for various different viral epidemics [16]. In case of coronaviruses, bats and rodents are found to be significant sources and reservoirs [17]. Likely, the coronavirus genomic RNA segments also transferred from bat and rodent species to humans or other mammals. Indeed, nCOVID-19 is the third encounter of the coronavirus, alongside SARS-CoV (China, 2003) and MERS-CoV (Saudi Arabia, 2013) in a short time interval. Interestingly, a molecular modeling prediction suggests that both SARS-CoV-2 and SARS-CoV viruses exhibit 79% homology at the nucleotide level and utilizes human ACE2 (Angiotensin Converting Enzyme 2) to bind their S1 spike protein [18]. However, transmission and mortality rates with SARS-CoV were found to be significantly lower as compared to SARS-CoV-2. This can be seen during the SARS-CoV epidemic, in the year of 2003, where 8000 deaths were reported [19, 20]. On the other hand, there is constant mutation and adaptation of the co-evolved CoVs S1 spike protein binding partner human ACE2 at the order, classes, and species level [21, 22]. Recent findings suggest the variable expression of human ACE2 is critical to the spread of the SARS-CoV-2 in the aging population [23]. Different studies suggest the coronavirus evolutionary relatedness with human ACE2 plays an important role, and hosts ACE2 switching could be a limit factor for COVID-19 pathogenesis majorly in elderly persons.

## 3. Immune senescence and SARS-CoV-2 infection

### 3.1. Cellular senescence, SASP and Aging

Cellular senescenece is the irreversible loss of cell proliferation and cell cycle growth arrest. It is mainly attributed to the DNA damage, cellular and metabolic stress [24]. The cells undergoing to senescence secrete increased amount of inflammatory factors such as chemokines (CXCL1, CXCL9, CXCL 10, CXCL11, CCL, CCL4), and cytokines (TNFα, IL1b, IL6, IL8, IL18 and IL12 cytokines), this phenotype of increased secretion of factors is termed as senescence-associated secretory phenotype (SASP)[25, 26]. Rodier et al observed that the persistent DNA damage in the senescent cells (SCs) lead to the increased secretion of cytokines [27]. The increased secretion of cytokines, termed as cytokine storm, found to exacerbate tissue damage and pathology in SARS-CoV-2 and other viral infections [28-31]. Further, Dong et al provided the direct evidence between senescence and viral infection severity, they found when senescence-accelerated mice infected with influenza A virus, there is significant reduction of cellular immunity leading to higher mortality rate and increased persistence of virus in the lungs [32]. Usually immune cells helps to clearance of senescent cells, however, during aging there is decline in immune surveillance leading to accumulation of senescent cells in several aging tissues and therefore, accumulation of SCs leads to induce SASPs and possess higher tissue damage [33-37]. On the other hand, decreased lymphoid lineage from aging hematopoietic stems cells are shown to provide an impaired antigen recognition, lessen memory CD8 T cell, and poor vaccine response to viral infection [38, 39]. Whereas abundantly differentiated myeloid cells provide weaken immune response and are shown to be incompetent in viral clearance in aging and underlying health condition individuals [40, 41].

### 3.2. Paradox of ACE2 expression and SARS-CoV-2 infection

Recent findings reveal that human Angiotensin-Converting Enzyme 2 (ACE2) is a distinct gateway for the entry of SARS-CoV-2 into cells. Studies from South Korea and Italy's epidemiologic data confers that the ACE2 expression declines with aging, leading a predisposed co-morbidity in older individuals with cardiovascular disease infected with COVID-19 [21, 42]. These datasets explain an apparent paradox; if ACE2 itself allows SARS-CoV-2 to enter in the cell, then how does the reduction of ACE2 expression exacerbate COVID-19 manifestation in older persons? Given the fact that hACE2 also plays an essential anti-inflammatory function converting angiotensin II to angiotensin 1-7 during RAS signaling in the cells. The increased RAS signaling leads to a decline in ACE2 expression and aging, leading to the induction of pro-inflammatory signaling and exacerbating the elderly's pathology. Likely, CD26, also known as dipeptidyl-peptidase IV (DPP4), serve as host receptor to recognize highly related MERS-CoV coronavirus. Coincidently, CD26 highly expresses on senescent profibrotic myofibroblast cells. The upregulation of CD26 expression on senescent cells provides an excellent opportunity to bind the MERS-CoV spike and to produce large amounts of inflammatory cytokines as a result of the senescence-associated secretory phenotype (SASP), IL-6 and other inflammatory cytokines [7, 43-46].

### 3.3. Toll-like receptors and SARS-CoV-2

Invading RNA virus particles are usually recognized by host innate Toll-like receptors (TLR) TLR 3, TLR7, TLR8, and TLR9, RIG-I, MDA5, and cGAS and trigger the series of the immune response in the respiratory mucosa. Followed by, Interferon Regulatory Factor 3 (IRF3), Nuclear Factor-κB (NF-κB) are major transcription factor to produce the type I Interferons (IFN-α/β) as well as a series of pro-inflammatory cytokines [47, 48]. Age-associated dysregulation of TLR expression and mediated signaling inflammatory shown to increased mortality of the elderly battling with the viral infection. Furthermore, the age-associated reduction of SARS-CoV-virus specific CD8 T memory cells in elderly mouse lungs exacerbates lung pathology to SARS-CoV infection [49]. SARS-CoV-virus specific CD8 T memory cells are shown to protect against SARS-CoV infection, emphasizing the host’s adaptive immune response toward COVID-19 clearance. Interestingly, CD26 also expresses CD8 T cells and helps to recognize MERS-CoV viruses. The decline expression of CD26 in aging could precede the deficit numbers of antigen-specific CD8 T cells in aged mice infected with SARS-CoV in the lungs [50].

### 3.4. Cytokine storm and SARS-CoV-2

Overwhelming cytokine storm also reported to exacerbates high fever and low blood pressure incur to underlying health condition COVID-19 elderly patients and succumbed them with acute respiratory distress syndrome [51]. Plasma cytokines and chemokines, including IL-1b, IL-2, IL-4, IL-10, IL-12, IL-13, IL-17, MCSF, MCP-1, MIP-1α, TNF-α, and IFN-γ majorly found in the immunopathology of COVID-19 patients [31]. The early detection of plasma cytokines is challenging for COVID-19 patients as it takes a few days before a cytokine storm is detectable. Late detection of the high surge of plasma cytokines leads to severe tissue damage in the lungs. Senescent cells under the aging process tend to produce enhanced protein synthesis but also produces increased SASP inflammatory mediators, required for the onset of chronic inflammation as well as disease manifestation. The magnitude of senescence led to a high surge of IL6, IL1b, IFNγ, C-reactive protein, and TNFα is reported in several epidemiological studies to lower airway and lung injury and risk for in COVID-19 elderly patients [4].

### 3.5. Autophagy and SARS-CoV-2

On the other hand, autophagy is the conserved selective degradation process that removes damaged cellular components during viral infections, cellular stress, and aging processes. Interestingly, during evolution, each virus has developed its own unique strategies to hijack and subvert the autophagy processes to immune evasion and their replication [52, 53]. Such as, the *Paramyxoviridae, Orthomyxoviridae, Togaviridae,* and *Herpesviridae* virus families are known to induce autophagy [54-63]. The studies shown the use of autophagy inhibitor, hydroxychloroquine for the inhibition of Zika virus transmission in pregnant mice which suggests the antiviral role of widely used drug in SARS-CoV-2 infection [64]. MERS-CoV viral replication have been shown to decrease autophagy regulator Beclin1 (BECN1), and autophagy agonist leads an at least 28,000-fold decrease of MERS-CoV replication [65]. Besides, autophagy persuading drugs, rapamycin, statins, carbamazepine, and metformin is known to have antiviral activities [66]. Growing evidence explicit that autophagy is unlikely to play a role in the replication of beta coronaviruses, MHV, and SARS-CoV and autophagy acts as a cellular defense to modulating SARS-CoV-2 and ACE2 interactions. Interestingly, the decline in the autophagic process has been described in aging and aging-associated diseases and raises concern if the decline in autophagy is involved in COVID-19 infection [53, 67-71]. A detailed underlying mechanism elucidating the autophagy function during SARS-CoV-2 infection in the elderly remains unknown and warrant further research.

## 4. Combat with COVID-19 infection with an underlying condition in aging

Recent evidence suggests that COVID-19 infection opportunistically targets the senescent lung cells of advanced elderly peoples. Mechanistically, senescent cells have increased propensity for protein synthesis, which allows a succeeding environment for efficient viral particle production. Senolytics compounds are used to eliminate cellular senescence and remove senescent cells. Senolytics compound Resveratrol (3,5,4-trihydroxy-trans-stilbene) found in grapes, increases the host’s autophagy induction by stimulating SIRT1 pathways in the cells [72, 73]. Additionally, Resveratrol function has been shown to alleviate several viral diseases. Tomatidine is another natural senolytics compound that exhibits antiviral properties for dengue virus serotypes and Chikangunya virus infection and shown to induce autophagy and alleviate lifespan in several organism model systems [74-76]. FDA approved autophagy agonist drugs like carbamazepine, rapamycin, statins, and metformin known to antiviral activities. Research purposes of these drugs and there potential impacts towards the SARS-CoV-2 infection have not been explored and warrant further investigation[77, 78].

New vaccine research aims to produce antibodies to block virus SARS-CoV-2 spike protein S1 subdomain interaction with host ACE2 receptor, and ultimately stop virus replication. Although developing a SARS-CoV-2 vaccine for older people is challenging, since the elderly historically do not respond as adequately to vaccines as a younger age group. Recent research has found several candidates for vaccines for SARS-CoV-2 in the animal models. However, an inadequate immune response coupled with a vaccine of low efficacy results in lung damage and high mortality of the animal [79-83]. Comprehensive research requires designing a compelling live or attenuated COVID-19 vaccine, and carefully look at whether they could provide long-term protection to COVID-19 infection in the elderly. Congruently, single transfusion of convalescent plasma obtained from recovering virus-infected patients gains recent attention to reduced pathology and mortality in SARS-CoV-2 morbidity in recent clinical trials [84]. Transfusion of convalescent plasma has also shown promising effects of protection in previous outbreaks such as SARS-CoV, MERS, Ebola, H1N1, and H5N1 avian influenza viruses [85-89]. However, plasma transfusion contains technical challenges of 3.7% clotting, 1.5% of cases of blood access difficulties, and 1.5% allergic reactions have been found. Plasma transfusion in the elderly population is not commonly used[90]. However, along with experienced staff, it may be a safe and efficient method to improve the outcome of elderly patients of SARS-CoV-2 morbidity. Plasma transfusion in the elderly population is not commonly used[90]. However, along with experienced staff, it may be a safe and efficient method to improve the outcome of elderly patients of SARS-CoV-2 morbidity.

High-throughput screening of new drug targets is recently emerging as the fastest way to find potential therapeutic molecules for the ongoing COVID-19 pandemic. Bioinformatic analysis of existing genomic information, protein codes, and pathological databases is compared with other coronaviruses, such as SARS-CoV and MERS-CoV, to screen molecules that may have a therapeutic effect on coronavirus. These approaches will enable the prediction of a variety of compounds that may inhibit novel coronavirus and could provide scientists with information on compounds that may be effective. The off-target drug is a significant drawback in making a broad-spectrum target for SARS-CoV-2 infection, and their side effects should not be underestimated. Subsequent *in vitro* and *in vivo* validation requires the measurement of efficacy and the antiviral effects of the final compound in the clinical treatment of COVID-19 infection.

## 5. Conclusion

Based on the recent frameworks, this review offers insight on a comprehensive overview of the COVID-19 pandemic, as well as plausible therapeutics for elderly people battling a COVID-19 infection. Epidemiological data suggests massive mortality and co-morbidities found in frail older people with underlying health conditions living in community-dwelling nursing homes assisted living facilities. The transmission of SARS-CoV-2 is more advanced than previous SARS-CoV and MERS-CoV outbreaks. The viral pathogen has developed strategies during co-evolving with the host to subvert the host immune system. Thereby, a clear understanding of the SARS-CoV-2 receptor binding domain protein and their binding partner human ACE2 expression is required in finding an active target to block SARS-CoV-2 binding and their entrance in the cell. Our phylogenetic analysis data suggests the host ACE2 also evolved order, classes, and species-level along with viral spike protein. However, variable switching ACE2 expression in-particular to elderly persons need to understand better, which could be a limit factor for COVID-19 pathogenesis.

Cellular senescence triggers a hyperimmune inflammatory response and causes high mortality in aging people. The dysregulation of the expression of toll-like receptors and impaired SARS-CoV-2 virus specific CD8 T memory cell response leads to disease exacerbation in elderly mice. The high surge of IL6, IL1b, IFNγ, C-reactive protein, and TNF-α senescence leads to lower airway and lung injury and increased risk for COVID-19 in elderly patients. The early detection of plasma cytokine storm is challenging, and late detection of the high surge of plasma cytokines led to severe tissue damage in the lungs. Given senescent cells tends towards enhanced protein synthesis, increased SASP inflammatory mediators, which would make senescent cells an ideal host target for increase viral replication. Senolytics usage has been proposed and could be promising in the treatment of COVID-19. Autophagy, on the other hand, and other cellular mechanisms targeting viral infection and replication are controversial and finding a common mechanism for COVID-19 infection needs further efforts to elucidate. Apart from pharmaceutical agents, modulating autophagy is adequate in controlling viral infections, although further study is required for the access of an autophagy modulator in COVID-19 infection. Neutralizing the ACE2 receptor, designing antibodies against ACE2 could better candidates for vaccination, although, a detailed clinical trial required for access to clinical outcome and side effects.

Overall, the field of gerontology has been overwhelmed by COVID-19, and prominent therapeutics are long-awaited. Therefore, for future studies, the underlying mechanism of immune senescence should be considered for useful drug invention or protective vaccination in the elderly.
